# Changes of bacterioplankton apparent species richness in two ornamental fish aquaria

**DOI:** 10.1186/2193-1801-2-66

**Published:** 2013-02-23

**Authors:** Nikolaos Vlahos, Konstantinos Ar Kormas, Maria G Pachiadaki, Alexandra Meziti, George N Hotos, Eleni Mente

**Affiliations:** 1 Department of Ichthyology & Aquatic Environment, School of Agricultural Sciences, University of Thessaly, Volos, 384 46 Greece; 2 Department of Aquaculture & Fisheries Management, Technological Educational Institute of Mesolonghi, Mesolonghi, 30 200 Greece

**Keywords:** Bacteria, 16S rRNA gene, Water column, Diversity, Ornamental, Fish, Aquaria

## Abstract

We analysed the 16S rRNA gene diversity within the bacterioplankton community in the water column of the ornamental fish *Pterophyllum scalare* and *Archocentrus nigrofasciatus* aquaria during a 60-day growth experiment in order to detect any dominant bacterial species and their possible association with the rearing organisms. The basic physical and chemical parameters remained stable but the bacterial community at 0, 30 and 60 days showed marked differences in bacterial cell abundance and diversity. We found high species richness but no dominant phylotypes were detected. Only few of the phylotypes were found in more than one time point per treatment and always with low relative abundance. The majority of the common phylotypes belonged to the Proteobacteria phylum and were closely related to *Acinetobacter junii*, *Pseudomonas* sp., *Nevskia ramosa*, *Vogesella perlucida, Chitinomonas taiwanensis, Acidovorax* sp., *Pelomonas saccharophila* and the rest belonged to the α-Proteobacteria, Bacteroidetes, Actinobacteria, candidate division OP11 and one unaffiliated group. Several of these phylotypes were closely related to known taxa including *Sphingopyxis chilensis, Flexibacter aurantiacus* subsp. *excathedrus* and *Mycobacterium* sp. Despite the high phylogenetic diversity most of the inferred ecophysiological roles of the found phylotypes are related to nitrogen metabolism, a key process for fish aquaria.

**Electronic supplementary material:**

The online version of this article (doi:10.1186/2193-1801-2-66) contains supplementary material, which is available to authorized users.

## Introduction

The spatial and temporal distribution of organisms is considered one of the first and most important steps in understanding the distribution of life on the planet. More specifically, knowledge of the distribution of multiple species in time and space within a shared habitat is valuable for understanding how an ecosystem functions (Konopka, 2009). For example, a stable microbial community structure is considered to be a critical factor for ecosystem resilience after disturbances and along biogeochemical cycles of elements and materials (Torsvik and Øvreås, 2002; Ramond et al. 2012). In microbial habitats, i.e., where microbes are the sole living organisms or hold the key ecophysiological roles, the changes in community structure are even more important. It is known that under stable conditions a few bacterial phylotypes are expected to dominate and/or few alterations in community structure occur until some environmental perturbations take place (Øvreås and Curtis, 2011). Despite intensive studies into the bacterioplankton community structure in natural marine and freshwater habitats (Bomberg et al. 2008; Kirchman, 2008; Barberan and Casamayor, 2010; Newton et al., 2011) and the use of artificial controlled systems for studying microbial community dynamics (e.g. Massana et al. 2001), engineered systems, e.g., fish aquaria, have been understudied regarding their bacterioplankton dynamics.

Little is known about the bacterioplankton communities’ structure and dynamics in ornamental fish aquaria. Most of the studies that have been conducted have focused either on the isolation of bacteria from fish tissues (e.g., Beran et al. 2006) or biofilters in the aquaria (Hovanec and DeLong, 1996; Burrel et al. 2001; Grommen et al. 2005; Sugita et al. 2005; Sauder et al. 2011). However, these systems harbour interesting bacterial communities that might contain antibiotic-resistant strains as a result of the water treatment process during commercial transportation of the fish (Trust and Whitby, 1976). Regarding the two species we examined, i.e. *Pterophyllum scalare* and *Archocentrus nigrofasciatus*, it is known that *P. scalare* is susceptible to mycobacteriosis (Lescenko et al. 2003), although mycobacteria are found in healthy fish as well (Beran et al. 2006). These two species are among the most popular species in the global ornamental fish market. Both cichlid species are omnivorous (Garcia-Ulloa and Gomez- Romero, 2005; Bernstein, 1980), carnivorous (Degani, 1993) and saprotrophs/detrivores (Crampton, 2008).

Small glass aquaria with recirculating systems that allow control of environmental conditions, disease, feeding and stocking densities are used to rear freshwater ornamental fish. However, food and water quality are among the most important factors shown to affect the growth of ornamental fish and optimal culture methods and techniques for current commonly traded and cultured species are still needed. There are 1300–2000 cichlid species worldwide that utilize various dietary sources and vary greatly in their dietary requirements (Knop and Moorhead, 2012). *P. scalare* and *A. nigrofasciatus* consume plankton, mosquito larvae, crustaceans (copepods), plants and worms (Soriano-Salazar and Hernadez-Ocampo,  2002). Under captivity in aquaria conditions, their feeding up to the larvae stage is restricted to macrozooplankton organisms such as *Daphnia, Moina* and *Artemia* nauplii (Lim and Wong, 1997). Artificial diets are also used, most frequently as flakes or pellets (Luna-Figueroa et al. 2000).

As a side project of a broader study on growth and food consumption of the freshwater ornamental fish *Pterophyllum scalare* and *Archocentrus nigrofasciatus*, we investigated the changes in bacterioplankton species composition during a 60 day growth experiment of the two species. We monitored the bacterial 16S rRNA gene diversity in order to elucidate whether (a) any dominant Bacteria can be found and (b) there are bacterial species that persist in the tanks. We chose the two tropical Cichlidae species as they have similar rearing conditions and, based on our previous expriments, they have similar growth rates despite their differences in food consumption.

## Materials and methods

### Rearing conditions

The rearing experiments were conducted at the laboratory of aquaculture and fisheries in the Technological Education Institution in Mesolongi, in Greece. Larvae were hatched from ovigerous females that had been grown in the laboratory under captivity. They were fed *ad libitum*, *Artemia* nauplii, (JBL Artemio Pur type, Germany) *Cyclops* and *Daphnia* (Ocean nutrition, Belgium) (Sorgeloos et al. 1986) for ten days and then with appropriately sized pellets and flakes containing 42% protein until day 30. A group of 20 chosen angelfish (*P. scalare,* 1.10 ± 0.5 g mean initial wet weight) and 20 convict cichlids (*A. nigrofasciatus,* 1.11 ± 0.11 g mean initial wet weight) were selected and transferred to four glass aquaria (10 fish per rearing units). Growth took place in 45 l glass aquaria (41 × 36 × 30.5 cm) filled with fresh water and with an independent recirculation system. The growth experiments were set up as a randomized complete block design with three blocks containing one replicate of the six treatments. Each block consisted of six 45 l glass aquaria (rearing tanks) with an independent recirculation system. They were hand fed 5% of their body weight three times per day for a period of 60 days with a commercial diet consisting of pellets and flakes. The feeding rate was adjusted every two weeks. The tanks were cleaned and uneaten food was removed every day. The fish weighed 2 g with no significant statistical differences (*P > 0.05*) between their final weights at the end of the trial.

During the trial, the water temperature was kept at 25°C or 20°C, the optimal growth temperatures for *P. scalare* and *A. nigrofasciatus*, respectively. Temperature was kept stable throughout the growth period by using air-conditioning and a stainless steel immersion heater located in the head reservoir. A false perforated plastic bottom was properly fitted in each aquarium and was covered with 6.5 Kg of lava grain to act as a filter bed substrate. The surface area of each filter bed was 1476 cm^2^. The water was continuously recycled through the filter bed using an air-lift pump with an adjusted flow of 5118 ml min^-1^ to yield a filtration speed of 3.88 cm min^-1^. The tanks were left for 24 hours in order to allow traces of chlorine in the tap water to be removed. One to two days after that, ca. 50 × 10^6^ nitrifying and denitrifying cells (Biodigest Probio, France) were added. The addition of these cells is expected to be low compared to the existing cell counts. The addition in the first three days of 50 × 10^6^ cells in 45 l of waters equals ca. 1,111 cells ml^-1^. On day 2 the bacterial abundance was between ca. 2.4 and 5.3 × 10^6^ cells ml^-1^ and on day 5 it was ca. 1.2 and 4.3 × 10^6^ cells ml^-1^, which renders the introduced 1,111 cells rather negligible. The tanks were sterilised at the start of the experiment, however, the observed cell counts were due to the aged tap water used for these rearing experiments.

An amount of 0.2 g solid NH_4_Cl was then dissolved in each aquarium to serve as the ammonia source (Vlahos et al. 2004). Approximately 10% of the water in each tank was replaced every day with fresh tap water. The aquaria were subjected to a photoperiod of 12 h:12 h light:dark, and the starting pH was 7.8 – 8.0. The concentrations of nitrate, nitrite and ammonia were measured every two or three days spectrophotometrically by using commercial kits (Hach Lange, USA). The dissolved oxygen concentrations and pH were measured with an HQ 40d multiprobe (Hach Lange, USA).

### Bacterial abundance and diversity

Bacterial cell abundance and 16S rRNA diversity were investigated at the beginning (0d), middle (30d) and end (60d) of the rearing period for each species from one tank for each of the species. To investigate the bacterioplankton diversity, we sampled only from one tank for each species in order not to disturb the major rearing experiment (e.g. water level reduction, possible water tank contamination from handling, etc.) which could mask the fish growth results. From each rearing tank, 50 ml of water was fixed with 2% formaldehyde final concentration and kept at 4°C in the dark. A subsample of 10–15 ml was filtered on black Nuclepore filters (pore size of 0.2 μm) and stained with DAPI (4',6-diamidino-2-phenylindole). After mounting the filters on glass slides, the cells were counted on an Axiostar (Zeiss) epifluorescence microscope at 1,000 × magnification (Porter and Feig, 1980). DAPI counts were counted thrice for every sampling time point from the same tank and the coefficient of variation was always less than 10% (data not shown). In parallel, 1–2 l of water was filtered on 47 mm diameter 0.2 μm pore-size polycarbonate filters of (Millipore, USA) under low vacuum pressure (≤150 mm Hg) and the filters were stored at −80°C. DNA was extracted using the UltraClean Soil DNA isolation kit (MoBio Laboratories, USA) according to the manufacturer’s protocol after slicing the filters with a sterile scalpel. DNA concentrations ranged from 7.6 – 17.8 ng μl^-1^ (A260/A280 ratios 1.87-2.13). Bacterial 16S rDNA was amplified using the bacterial primers GM3 (5′- AGAGT TTGATCMTGGC-3′) (Muyzer et al., 1995) and 1390r (5′-TGTACACACCGCCCGTC-3′) or GM4 (5′-TACCTT GTTACGACTT-3′) (Lane at al., 1991). The PCR included an initial denaturation step at 94°C for 1 min followed by 25 to 29 cycles consisting of denaturing at 94°C for 45 s, annealing at 44°C for 45 s, and elongating at 72°C for 2 min with a final 7-min elongation step at 72°C after the final cycle. The number of cycles was determined for each sample after cycle optimisation. PCRs were repeated with different cycle numbers, and the lowest number of cycles that gave a positive signal was then used for cloning and sequencing in order to avoid differential representation of the 16S rDNA genes with low and high copy numbers (Spiegelman et al., 2005).

Polymerase chain reaction products were visualized on a 1% agarose gel under ultraviolet light, the bands were excised, and the PCR products were extracted with the PureLink Quick Gel Extraction Kit (Invitrogen Corporation, USA) following the manufacturer’s protocol. The PCR products were cloned using the TOPO TA for sequencing cloning kit (Invitrogen Corporation, USA) and electrocompetent cells according to the manufacturer’s specifications. For each sample and each gene, randomly picked clones with inserts of the expected length were analysed. Clones were grown in liquid LB medium with kanamycin and their plasmids were purified using the NucleoSpin Plasmid QuickPure kit (Macherey-Nagel GmbH & Co. KG, Germany) for DNA sequencing.

### Sequencing and phylogenetic analysis

Sequence data were obtained by Macrogen Inc. (South Korea) using capillary electrophoresis and the BigDye Terminator kit (Applied Biosystems Inc., USA) with the primers M13F (−20) and M13R. Each sequence read was approximately 900 bp, and for each individual clone, forward and reverse reads were assembled. Chimeric sequences were checked by comparing neighbour-joining trees made of the first and second halves of all sequences. Sequences with different groupings between the first and second halves were then checked using the Pintail program (http://www.bioinformatics-toolkit.org/Web-Pintail/).

The closest relatives for all retrieved sequences were determined by comparison using the BLAST function (http://www.ncbi.nlm.nih.gov/ BLAST/). Automatic alignment against sequences from their closest relatives was performed using SILVA (http://www.arb-silva.de/aligner/). Phylotypes were defined as sequences showing ≥98% homology to each other. Phylogenetic trees were constructed by the neighbour-joining method using the Jukes-Cantor correction. Bootstrap analyses for 1000 replicates were performed to assign confidence levels to the tree topology using the MEGA4 software (Tamura et al., 2007). The sequences of unique phylotypes found in this study have GenBank numbers JX105530 - JX105733.

The clone library coverage was calculated using the equation C = [1 - (n_i_/N)]x100, where n_i_ is the number of phylotypes and N is the number of 16S rRNA sequences examined (Good, 1953; Kemp and Aller, 2004).

## Results

### Physical and chemical parameters

**Figure 1:**
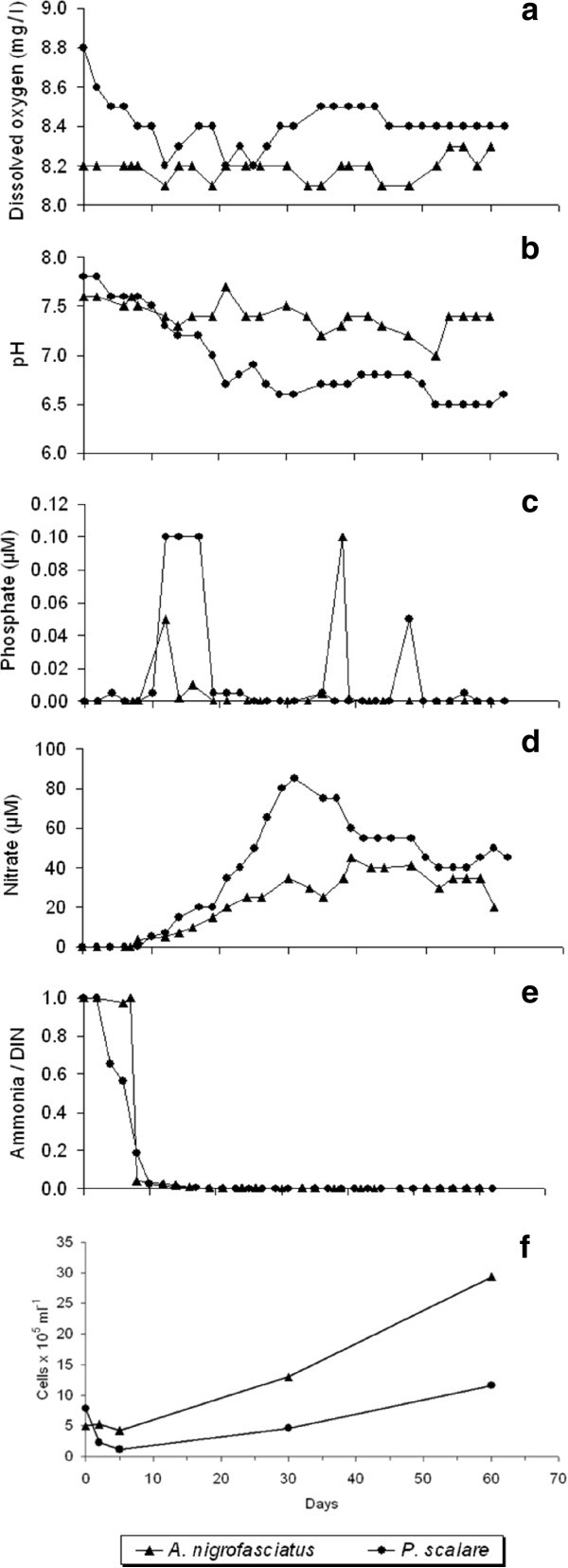
**Temporal changes in (a) dissolved oxygen, (b) pH, (c) phosphate, (d) nitrate, (e) ammonia/DIN and (f) bacterial abundance (cells × 10**^**5**^**ml**^**-1**^**) in the water column of the rearing tanks of*Archocentrus nigrofasciatus*and*Pterophyllum scalare.*** DIN: dissolved inorganic nitrogen, i.e. total concentrations of nitrate, nitrite and ammonia.

Temperature remained stable at the desired values throughout the rearing periods for both species. Although the concentrations of dissolved oxygen (Figure 1) showed different fluctuation patterns for the two rearing treatment (8.1 – 8.3 and 8.2 – 8.8 mg l^-1^ for *A. nigrofasciatus* and *P. scalare*, respectively), no limiting concentrations were detected. Variations in the pH (Figure 1) were also different, especially after the first 10 days (7.0 – 7.7 and 6.5 – 7.8 for *A. nigrofasciatus* and *P. scalare*, respectively). Phosphate levels (Figure 1) showed little variation (0.000 – 0.001 μM) for both species, but the highest values were obtained at different time points for the two species. Regarding nitrogen-containing nutrients (Figure 1), no ammonia was detected after the first six to eight days of the experiment but nitrate levels increased after this time point.

### Bacterial abundance and diversity

The water column cell counts increased until the end of the rearing period for both species (Figure 1). The initial cell abundance was comparable in both treatments (0.5 and 0.8 × 10^6^ cells ml^-1^ for *A. nigrofasciatus* and *P. scalare*, respectively) and was followed by an initial decrease in the *P. scalare* tanks. At the end of the experiment, the bacterial cell abundance reached 2.9 × 10^6^ and 1.2 × 10^6^ cells ml^-1^ for *A. nigrofasciatus* and *P. scalare*, respectively.

**Figure 2:**
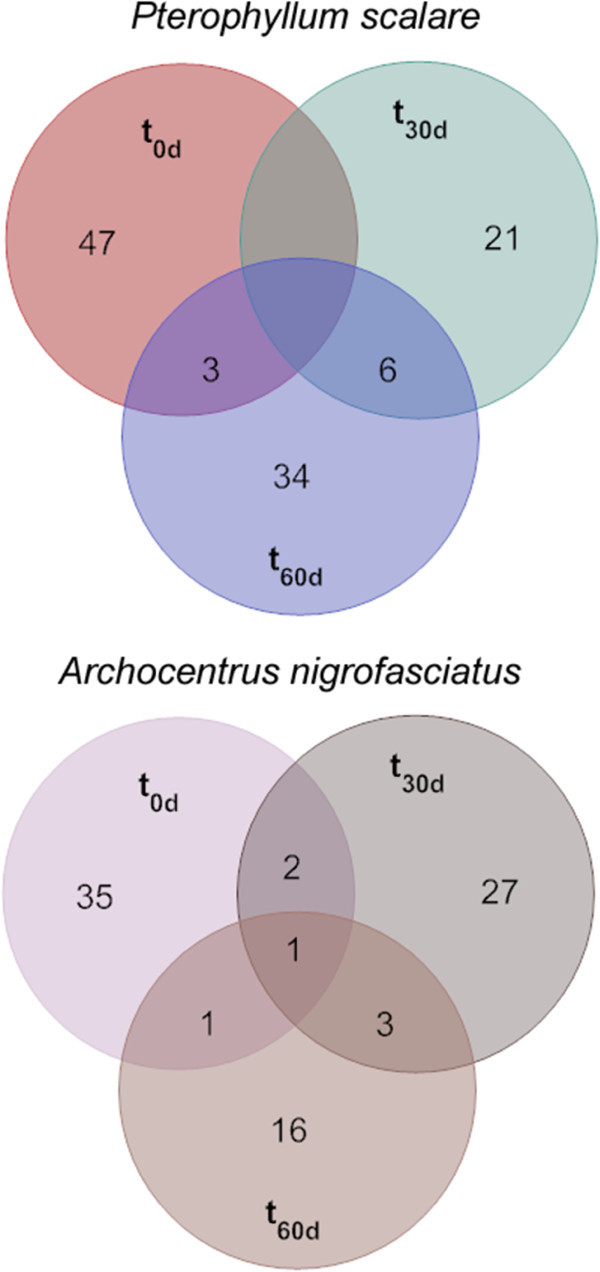
**Venn diagrams of the common phylotypes occurring in the water column of the rearing tanks of*Archocentrus nigrofasciatus*and*Pterophyllum scalare*at the beginning (t**_**0d**_**), middle (t**_**30d**_**) and the end (t**_**60d**_**) of the growth period.**

For each clone library we analysed 41–72 clones, which corresponded to 21 – 50 unique phylotypes (Figure 2). The level of clone coverage showed that the estimated species richness of the samples is high (Additional file 1: Figure S1). This, along with the lack of any dominant phylotype (i.e., the most abundant phylotypes reached 8.6% and 13.9% for *A. nigrofasciatus* and *P. scalare*, respectively) and the numerous singletons or doubletons, suggest the presence of highly diverse communities that have not been fully revealed using the current methodology.

In both treatments, the highest number of phylotypes occurred at 0day but the lowest occurred at 60days and 30days for *A. nigrofasciatus* and *P. scalare*, respectively For each treatment, only two to six phylotypes were found in more than one clone library (Figure 2) but their relative abundance was low in most cases. For *A. nigrofasciatus*, phylotype T0-An-20C-58 occurred at all three time points, but no common phylotypes were detected for *P. scalare* between 30 days and 60 days. No common phylotypes were found to dominate any of the sampling points in each of the aquaria (Additional file 1: Table S1). There were no common phylotypes identified between the aquaria of the two fish species.

**Figure 3:**
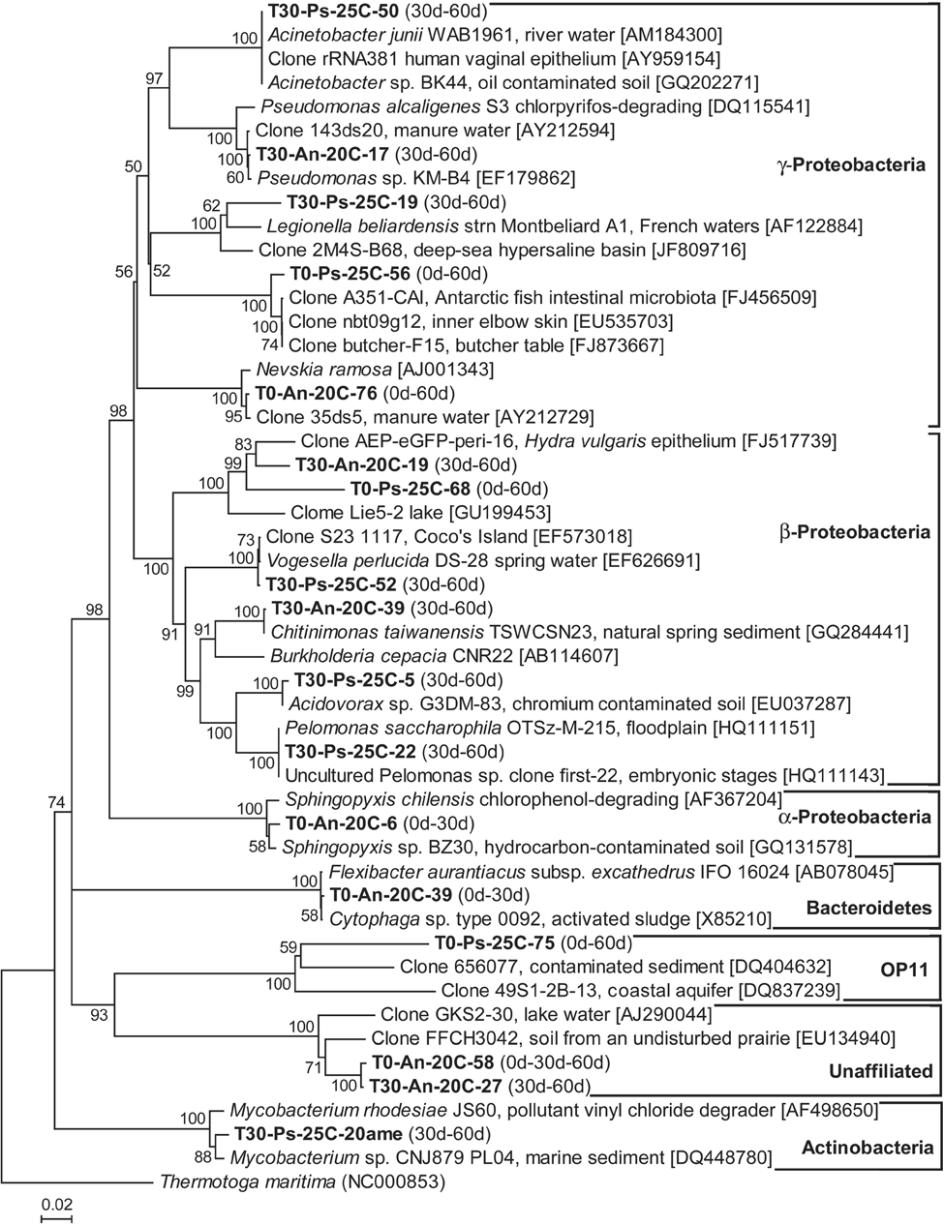
**Phylogenetic tree of the common Bacteria (in bold) occurring in the water column of the rearing tanks of*Archocentrus nigrofasciatus*and*Pterophyllum scalare*at the beginning (0d), middle (30d) and the end (60d) of the growth period.** The tree of the Bacteria 16S rRNA gene phylotypes (ca. 1,500 bp, 1013 positions) was based on the neighbour-joining method as determined by distance using Kimura’s two-parameter correction. One thousand bootstrap analyses (distance) were conducted, and percentages ≥50% are indicated at nodes. Numbers in brackets are GenBank accession numbers. Scale bar represents 2% estimated distance.

The phylogenetic analysis of the common phylotypes (Figure 3) revealed that the majority belonged to the Proteobacteria. These proteobacterial phylotypes were closely related to known taxa: *Acinetobacter junii, Pseudomonas* sp., *Nevskia ramosa, Vogesella perlucida, Chitinomonas taiwanensis, Acidovorax* sp., and *Pelomonas saccharophila*. The rest of the phylotypes belonged to the α-Proteobacteria, Bacteroidetes, Actinobacteria, candidate divison OP11 and one unaffiliated group. A subset of these phylotypes was closely related to known taxa including *Sphingopyxis chilensis*, *Flexibacter aurantiacus* subsp. *excathedrus* and *Mycobacterium* sp.

For both samples, the remaining phylotypes, occurred in only one clone library (Additional; file 1: Figure S2, S3) and belonged to the α-, β- γ- δ- and unaffiliated Proteobacteria, Fusobacteria, Bacteroidetes, Actinobacteria, Firmicutes and unaffiliated phyla. In the *A. nigrofasciatus* samples, phylotypes belonging to the Deinococcus-Themus, Planctomycetes, Verrucomicrobia phyla and some plastid-related clades were also found.

## Discussion

In this study, we showed that the bacterial communities developed in the water column under controlled rearing conditions in two ornamental fish aquaria were characterised by high species richness but low relative abundance. The coverage of the clone libraries was low due to the high number of rare species (i.e., species with ≤0.1% relative abundance). In tapwater samples (e.g., Kormas et al. 2010; Poitelon et al. 2010; Revetta et al. 2010) or drinking water reservoirs (e.g., Simek et al. 2001; Lymperopoulou et al. 2012) this quantity of sequences is adequate to illustrate the dominant members of the bacterial community. In our study, the coverage was only satisfactory for the 60days in the *A. nigrofasciatus* sample (Additional file 1: Figure S1), however, there were still no dominant phylotypes revealed.

Of all the measured parameters, pH and nitrate showed such fluctuating patterns that could affect the bacterial species community structure. The pH varied markedly for *A. nigrofasciatus* but not for *P. scalare* at the three sampling points. Nitrate showed the opposite pattern. Nitrate levels in *P. scalare* peaked on day 30 and then fell to almost half this value at day 60. For *A. nigrofasciatus*, no clear peak occurred and the nitrate levels never reached the ones for *P. scalare*. Despite these differences, both treatments showed little overlapping phylotypes between sampling points, rendering their presence independent from the changes of these parameters or at least with a time lag which was not detected at the 30 day intervals used in this study.

Nitrification must have been taking place in both treatments because ammonia was not detectable after the first six to eight days, and after that, nitrogen increased until the end of the experiment. Because nitrate and phosphate are considered major limiting factors for the growth of microorganisms in the aquatic environment, it is possible that the increased nitrate concentrations could sustain the observed increasing cell abundance until the end of the experiment while favouring, thus, the dominance of some nitrate reducers. Phosphate is also expected to be turned over fast i.e., it is used immediately which is why its levels remained below the detection limit.

It has recently been suggested that although the bacterial diversity and community structure can be highly variable between two communities, the functional redundancy of these different species can be high (Burke et al. 2011). Although there was no clear dominance of specific phylotypes in the current study, most of the common phylotypes were closely related to species that reduce nitrate reduction, most likely as a result of the increased nitrate concentrations after the first days of the experiment. Phylotype T0-An-20C-76 is closely related to *Nevskia ramosa*. This species grows optimally at 20 – 25°C (Brenner et al. 2005) at air-freshwater interfaces (Pladdies et al. 2004) or in drinking water biofilms (Keinänen-Toivola et al. 2006). Another phylotype (T30-Ps-25C-52) is a *Vogesella perlucida*-like bacterium that can perform nitrate reduction at 4–45°C, with 0–2% NaCl and at pH 7–9 (optimal growth at 30°C, 0.5% NaCl and pH 7.5) (Chou et al. 2008). Phylotype T0-An-20C-39 is related to *Burkholderia cepacia*, a species known to fix N_2_ but also a possible pathogen causing cystic fibrosis (Brenner et al. 2005. Phylotype T0-An-20C-39 has been found only in the posterior gut of semi-intensively cultured tilapia, *Oreochromis niloticus* (Molinari et al. 2003), and the Chilean freshwater-farmed Atlantic salmon (Miranda and Zemelman 2002), but it has been suggested to be involved in fish pathogenicity (e.g., catfish *Clarias gariepinus* fingerlinks, Nzeako et al. 2001). Consequently, it is very likely that this phylotype originated from the fish intestine suggesting that fish aquaria are sources of faecal microbes. However, its natural occurrence cannot be excluded as it also occurs in natural streams (Santmire and Leff 2006) and has been positively correlated to nitrate concentrations (Olapade et al. 2005). Thus, phylotype T0-An-20C-39 could contribute to the nitrification of the tank. Phylotype T30-Ps-25C-22 is related to *Pelomonas saccharophila* (Xie and Yokota 2005) and is also a potential nitrate reducer, along with T30-Ps-25C-5, an *Acidovorax* sp. group that contains denitrifiers (Brenner et al. 2005). Phylotype T0-An-20C-6 is most likely a *Flexibacter aurianticus*, which known to grow at 10-25°C and can reduce nitrate to nitrite (Brenner et al. 2005). The *Sphingopyxis* (synonymous with *Sphingomonas*)-like phylotype T0-An-20C-6 is also a known nitrate reducer (Godoy et al. 2003).

Bacterial abundance continued to increase until the end of the experiment due to the lack of considerable grazing pressure (Sherr and Sherr, 2002) evidenced by our failure to observe any nanoflagellates (i.e., always below detection limit) (Kormas, unpublished data). In batch (closed) cultures, the increase in bacterial cell numbers is usually attributed to few dominant species, but this was not the case in our study. Not only were there no dominant species, but very few of the ones that existed at day 30 and day 0 appeared in day 60 and high numbers of rare phylotypes were found.

Usually, high numbers of rare phylotypes are expected in more variable environments (Reid and Buckley, 2011). This number is decreased with the concomitant dominance of a few taxa in more stable habitats with less prominent changes in their prevailing conditions (Øvreås and Curtis, 2011). Possible ecophysiological mechanisms that may retain a high number of rare taxa include the following: (a) low metabolic or growth rates, (b) antagonism/allelopathy, (c) balance between growth and removal rates and (d) specialization in substrates or habitats (Pedrós-Alió, 2012). Regarding the metabolic rates of the inferred phylogenies, the prevailing conditions (i.e., temperature, pH, salinity) in the tanks fall in the range required by most of the common phylotypes (see above). Antagonism in the bacterioplankton of the tanks is expected to be high, and this could cause a lack of dominant phylotypes. Because we used closed systems, no new species could be introduced. However, the tanks were not isolated from the air and airborne bacteria could be introduced in the tanks, although their successful establishment remains doubtful and unknown. Top-down control was negligible as we did not observe any bacterial grazers, i.e. heterotrophic nanoflagellates or larger protozoa (Kormas, personal observations), but the effect of viral infection and lysis cannot be ruled out (Wommack and Colwell, 2000). The remaining factor that could cause ever-changing conditions in the water column of the tank is the quality and quantity of carbon sources. Carbon substrates are expected to originate from aquafeed leftovers and excretions from fish. The quality, quantity and lability of excreted carbon from fish, currently is not well known and this carbon pool remains a black box. Nevertheless, the production rate of dissolved and particulate nitrogenous waste by farmed salmon has been estimated (Davies 2000; Mente et al., 2006). Recently, the high or low sea bream (*Sparus aurata*) density in aquaculture cages was suggested to cause significant differences in bacterial cell numbers in response to higher or lower amounts of excreted material from the fish (Mente et al., 2012). The development of commercial rearing facilities that are in line with advances in research for optimal culture conditions will facilitate the further growth of sustainable ornamental aquaculture.

## Electronic supplementary material

Additional file 1 Table S1.: Changes in the relative abundance of the water column common phylotypes, at 0, 30 and 60 days, between samplings in the *Archocentrus nigrofasciatus* and *Pterophyllum scalare* rearing tanks. **Figure S1.** Bacteria clone library coverage based on Good’s C estimator from the water of *Archocentrus nigrofasciatus* and *Pterophyllum scalare* rearing tanks at the beginning (0 d) middle (30 d) and end (60 d) of the growth experiment. **Figure S2.** Phylogenetic tree of the Bacteria 16S rRNA gene phylotypes (ca. 1,500 bp, 1013 positions), excluding the ones that were found in more than one clone library, in the water column of *Archocentrus nigrofasciatus* rearing tank. The tree was based on the neighbour-joining method as determined by distance using Kimura’s two-parameter correction. Numbers of identical (≥98% sequence similarity) phylotypes of the total phylotype number in sample are shown in parentheses. One thousand bootstrap analyses (distance) were conducted, and percentages ≥50% are indicated at nodes. Numbers in brackets are GenBank accession numbers. Scale bar represents 2% estimated distance. **Figure S3.** Phylogenetic tree of the Bacteria 16S rRNA gene phylotypes (ca. 1,500 bp, 1013 positions), excluding the ones that were found in more than one clone library, in the water column of *Pterophyllum scalare* rearing tank. The tree was based on the neighbour-joining method as determined by distance using Kimura’s two-parameter correction. Numbers of identical (≥98% sequence similarity) phylotypes of the total phylotype number in sample are shown in parentheses. One thousand bootstrap analyses (distance) were conducted, and percentages ≥50% are indicated at nodes. Numbers in brackets are GenBank accession numbers. Scale bar represents 2% estimated distance. (DOC 688 KB)
