# Supplementary material for: Changes of bacterioplankton apparent species richness in two ornamental fish aquaria
Source: Springerplus. 2013 Feb 23;2:66. doi: 10.1186/2193-1801-2-66 (PMC3601261; doi:10.1186/2193-1801-2-66)
Supplement: Supplementary file 1 — Additional file 1 Table S1.: Changes in the relative abundance of the water column common phylotypes, at 0, 30 and 60 days, between samplings in the Archocentrus nigrofasciatus and Pterophyllum scalare rearing tanks. Figure S1. Bacteria clone library coverage based on Good’s C estimator from the water of Archocentrus nigrofasciatus and Pterophyllum scalare rearing tanks at the beginning (0 d) middle (30 d) and end (60 d) of the growth experiment. Figure S2. Phylogenetic tree of the Bacteria 16S rRNA gene phylotypes (ca. 1,500 bp, 1013 positions), excluding the ones that were found in more than one clone library, in the water column of Archocentrus nigrofasciatus rearing tank. The tree was based on the neighbour-joining method as determined by distance using Kimura’s two-parameter correction. Numbers of identical (≥98% sequence similarity) phylotypes of the total phylotype number in sample are shown in parentheses. One thousand bootstrap analyses (distance) were conducted, and percentages ≥50% are indicated at nodes. Numbers in brackets are GenBank accession numbers. Scale bar represents 2% estimated distance. Figure S3. Phylogenetic tree of the Bacteria 16S rRNA gene phylotypes (ca. 1,500 bp, 1013 positions), excluding the ones that were found in more than one clone library, in the water column of Pterophyllum scalare rearing tank. The tree was based on the neighbour-joining method as determined by distance using Kimura’s two-parameter correction. Numbers of identical (≥98% sequence similarity) phylotypes of the total phylotype number in sample are shown in parentheses. One thousand bootstrap analyses (distance) were conducted, and percentages ≥50% are indicated at nodes. Numbers in brackets are GenBank accession numbers. Scale bar represents 2% estimated distance. (DOC 688 KB) [file 40064_2012_139_MOESM1_ESM.doc]

**Changes in bacterioplankton apparent species richness in two ornamental fish aquaria**

Nikolaos Vlahos, Konstantinos Ar. Kormas*, Maria G. Pachiadaki, Alexandra Meziti, George N. Hotos, Eleni Mente

Supplementary material

Submitted to “SpringerPlus”

* Corresponding author: Tel.: +30-242-109-3082, Fax: +30-242-109-3157, E-mail: kkormas@uth.gr

**Table S1**. Changes in the relative abundance of the water column common phylotypes, at 0, 30 and 60 days, between samplings in the *Archocentrus nigrofasciatus* and *Pterophyllum scalare* rearing tanks.

| Species | 0-30 | 30-60 | 0-60 | 0-30-60 |
| --- | --- | --- | --- | --- |
| *Pterophyllum scalarae* | - | T30-Ps-25C-50  1.6% 🡪 3.8%  T30-Ps-25C-5  1.6% 🡪 3.8%  T30-Ps-25C-19  1.6% 🡪 3.8%  T30-Ps-25C-52  1.6% 🡪 5.7%  T30-Ps-25C-22  1.6% 🡪 1.9%  T30-Ps-25C-20ame  1.6% 🡪 3.8% | T0-Ps-25C-75  2.8% 🡪 3.8%  T0-Ps-25C-56  1.4% 🡪1.6%  T0-Ps-25C-68  1.4% 🡪 1.6% | - |
|  |  |  |  |  |
| *Archocentrus nigrofasciatus* | T0-An-20C-39  5.2% 🡪 4.9%  T0-An-20C-6  3.4% 🡪 2.4%  T0-An-20C-58  1.7% 🡪 4.9% | T30-An-20C-27  4.9% 🡪 2.2%  T30-An-20C-39  2.4% 🡪 8.9%  T30-An-20C-19  2.4% 🡪 2.2%  T30-An-20C-17  2.4% 🡪 26.7% | T0-An-20C-58  1.8% 🡪 2.2%  T0-An-20C-76  1.8% 🡪 2.2% | T0-An-20C-58  1.7% 🡪 4.9% 🡪 2.2% |


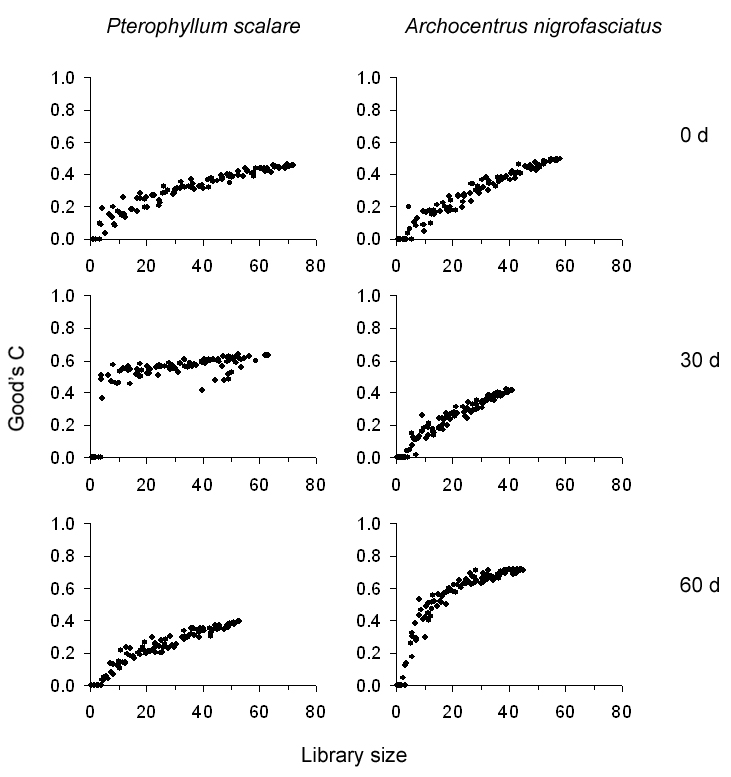


**Figure S1**. Bacteria clone library coverage based on Good’s C estimator from the water of *Archocentrus nigrofasciatus* and *Pterophyllum scalare* rearing tanks at the beginning (0 d) middle (30 d) and end (60 d) of the growth experiment.

**Figure S2**. Phylogenetic tree of the Bacteria 16S rRNA gene phylotypes (ca. 1,500 bp, 1013 positions), excluding the ones that were found in more than one clone library, in the water column of *Archocentrus nigrofasciatus* rearing tank. The tree was based on the neighbour-joining method as determined by distance using Kimura’s two-parameter correction. Numbers of identical (≥98% sequence similarity) phylotypes of the total phylotype number in sample are shown in parentheses. One thousand bootstrap analyses (distance) were conducted, and percentages ≥50% are indicated at nodes. Numbers in brackets are GenBank accession numbers. Scale bar represents 2% estimated distance.

**
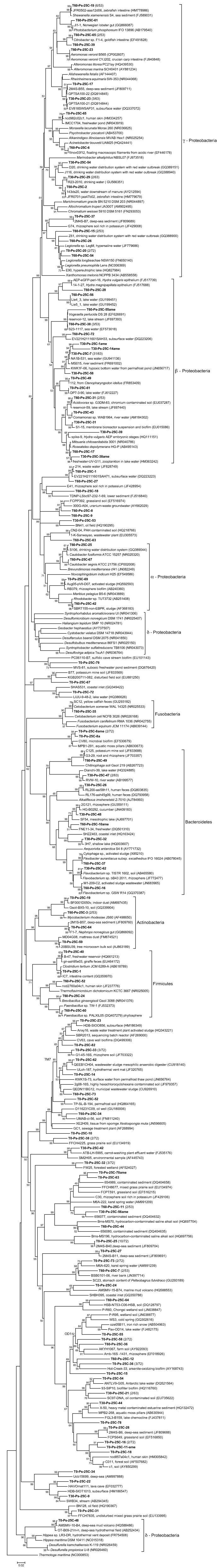
**

**Figure S3**. Phylogenetic tree of the Bacteria 16S rRNA gene phylotypes (ca. 1,500 bp, 1013 positions), excluding the ones that were found in more than one clone library, in the water column of *Pterophyllum scalare* rearing tank. The tree was based on the neighbour-joining method as determined by distance using Kimura’s two-parameter correction. Numbers of identical (≥98% sequence similarity) phylotypes of the total phylotype number in sample are shown in parentheses. One thousand bootstrap analyses (distance) were conducted, and percentages ≥50% are indicated at nodes. Numbers in brackets are GenBank accession numbers. Scale bar represents 2% estimated distance.
